# CTA imaging characteristics and endovascular therapy effect in patients with large ischemic stroke: a prospective cohort study

**DOI:** 10.1186/s42466-026-00492-6

**Published:** 2026-04-17

**Authors:** Wei Deng, Xiaojun Zhu, Jun Han, Yanli Sun, Guangxian Nan

**Affiliations:** 1 Department of Neurology, China-Japan Union Hospital of Jilin University, No.126 Xiantai Avenue, Erdao District, Changchun, 130000 Jilin China; 2 Department of General Practice, Xiangyang No.1 People’s Hospital, Hubei University of Medicine, No. 15 Jiefang Road, Fancheng District, Xiangyang, Hubei 441000 China; 3 Department of Neurology, Xiangyang No.1 People’s Hospital, Hubei University of Medicine, No. 15 Jiefang Road, Fancheng District, Xiangyang, Hubei China; 4 Department of Endocrinology, The First Affiliated Hospital of Anhui Medical University, Hefei, Anhui China

**Keywords:** Endovascular Therapy, Large Ischemic Stroke, Computed Tomography Angiography, Clinical outcomes

## Abstract

**Background:**

Although landmark trials showed favorable outcomes with endovascular therapy (EVT) for large ischemic stroke, imaging protocols for patient inclusion varied. This study aims to explore the interaction between computed tomography angiography (CTA) imaging characteristics and the association of EVT with outcomes in real-world clinical practice.

**Methods:**

This was a subanalysis of a prospective cohort study that enrolled patients with large vessel occlusion and ASPECTS 0–5 from 38 stroke centers across China between November 2021 and February 2023. Based on admission CTA imaging, the occlusion site, Tan score, cortical vein opacification score (COVES), internal cerebral veins (ICV), and clot burden score were assessed. The primary outcome was the 90-day modified Rankin Scale (mRS). Treatment effects across subgroups based on CTA imaging characteristics were analyzed using ordinal regression analysis, and interaction analysis was performed to assess whether these characteristics modified the effect of EVT on 90-day mRS.

**Results:**

Among the 460 patients (255 male, 55.4%), the EVT group was younger than SMT group (67 years [IQR, 58–77] vs. 72 years [IQR, 65–79]). COVES and Tan score significantly modified the association between EVT and 90-day mRS (*P* < 0.001 and *P* = 0.023 for interaction, respectively), with a larger effect size observed in patients with COVES 3–6 (adjusted common odds ratio [acOR], 2.43 [95% CI, 1.39–4.27]; *P* = 0.002) and Tan score 2–3 (acOR, 2.78 [95% CI, 1.24–6.24]; *P* = 0.013). In contrast, occlusion site, ICV score, and clot burden score did not modify the effect of EVT.

**Conclusion:**

Better arterial and venous collaterals in patients with large ischemic stroke are associated with favorable outcomes and may modify the effect of EVT. This analysis provides preliminary evidence supporting the role of baseline CTA imaging in guiding EVT selection for these patients.

**Supplementary Information:**

The online version contains supplementary material available at 10.1186/s42466-026-00492-6.

## Introduction

Recently, five randomized clinical trials have suggested that patients with large ischemic stroke can benefit from endovascular therapy (EVT), with most of these studies not requiring advanced imaging for patient inclusion [1–5]. Earlier studies have shown that, in both early and extended time windows, patients selected for EVT using advanced imaging have similar functional outcomes to those selected with noncontrast computed tomography (NCCT) or CT angiography (CTA) [6–8]. The Multicenter Randomized Clinical Trial of Endovascular Treatment of Acute Ischemic Stroke in the Netherlands for Late Arrivals (MR CLEAN-LATE) and a multinational cohort study by Nguyen et al. both demonstrated that EVT is efficacious and safe for large vessel occlusion (LVO) patients selected using CTA imaging in the extended time window [9, 10]. Furthermore, a meta-analysis found that whether patients were selected based on NCCT and CTA or advanced imaging in large-core stroke, there was no significant impact on procedural or patient outcomes [11]. Therefore, the role of time- and resource-consuming advanced imaging might be questioned in LVO patients with large ischemic stroke undergoing EVT.

CTA is broadly available and commonly used in real-world clinical practice, primarily for identifying the arterial occlusion site. Moreover, CTA imaging provides valuable insights into collateral status and clot burden, which are important characteristics that serve as strong predictors of clinical outcomes [12]. Internal carotid artery occlusion, poor arterial or venous collaterals, and a large clot burden are associated with ischemic core growth, early neurological deterioration, an increased risk of intracranial hemorrhage, and poorer functional outcomes after undergoing EVT [12–17]. However, evidence on the utility of CTA imaging characteristics for guiding patient selection for EVT in large ischemic stroke remains lacking. In this subanalysis of a prospective cohort study, we comprehensively describe CTA imaging characteristics, examined their interaction with EVT effects, and evaluated their prognostic associations with clinical outcomes in patients with large ischemic stroke.

## Methods

### Study population

This study is a subanalysis of a nationwide prospective cohort study involving 38 comprehensive stroke centers in China, which assessed the effectiveness and safety of EVT versus SMT in patients with large ischemic stroke between November 1, 2021, and February 8, 2023. [18] The study was registered in the Chinese Clinical Trial Registry (http://www.chictr.org.cn; ChiCTR2100051664), and the study protocol was approved by the ethics committees of the Second Affiliated Hospital of the Army Medical University and participating hospitals. Signed informed consent was obtained from all patients or their legally authorized representatives. This study followed the Consolidated Standards of Reporting Trials (CONSORT) reporting guideline.

The main inclusion criteria were as follows: patients aged 18 years or older; diagnosis of acute ischemic stroke (AIS) due to anterior circulation LVO (internal carotid artery [ICA], M1, or M2 segments of the middle cerebral artery [MCA]); presence of a large ischemic stroke on NCCT (Alberta Stroke Program Early CT Score [ASPECTS] 0–5); completion of baseline head CTA at admission; and presentation within 24 h of stroke onset or last known well. The main exclusion criteria were premorbid modified Rankin Scale (mRS) > 2, a lack of follow-up information at 90 days or baseline critical data, and/or a serious, advanced, or terminal illness that was not related to AIS.

### Imaging analysis

We assessed five imaging characteristics on admission CTA. All imaging data were analyzed by an independent core laboratory, where two experienced investigators (Dr Lai and Dr Deng), blinded to clinical outcomes, independently reviewed the scans. Discrepancies were resolved by consensus after adjudication by a third investigator (Dr Tian).

First, the occlusion site was determined on CTA imaging and classified into the ICA, M1 segments of the MCA, or M2 segments of the MCA. Second, arterial collaterals were assessed on single-phase CTA using the modified Tan scale and categorized as unfavorable (0–1) or favorable (2–3) [19]. Third, superficial venous collaterals were evaluated on single-phase CTA using the COVES, which grades the opacification of the vein of Labbé, sphenoparietal sinus, and superficial middle cerebral vein (0 = absent, 1 = partial, 2 = complete). A total score of 3–6 was considered favorable, whereas 0–2 was deemed unfavorable [20]. Fourth, deep venous collaterals were assessed using the ICV score, with a score of 2 classified as favorable and 0–1 as unfavorable. Finally, clot characteristics were assessed on CTA using the clot burden score, ranging from 0 (all anterior circulation vessels occluded) to 10 (no large-vessel occlusion), with lower scores indicating higher clot burden and worse prognosis [21].

### Outcomes measurements

The primary outcome was 90-day mRS, analyzed as a shift toward better functional outcomes, assessed via structured telephone interview by a blinded research nurse. Secondary outcomes included functional independence (mRS 0–2), favorable outcome (mRS 0–3), the proportion of patients with mRS 0–4 at 90 days, symptomatic intracranial hemorrhage (sICH, classified by the Heidelberg Bleeding Classification) [22], any intracranial hemorrhage within 48 h, and 90-day mortality.

### Statistical analysis

Continuous variables with a normal distribution are expressed as mean ± SD, while those that do not follow a normal distribution are presented as median and interquartile range (IQR). Categorical variables are presented as frequency and percentage. For univariate comparisons, the Kruskal-Wallis test or Mann-Whitney U test was used for continuous variables, and the χ² test or Fisher’s exact test was applied to categorical variables.

The primary objective was to assess the association between EVT and 90-day mRS across subgroups defined by CTA imaging characteristics. Effect sizes were estimated using ordinal regression and expressed as adjusted common odds ratios (acORs) with 95% confidence intervals (CIs), adjusting for age, sex, diabetes, hypertension, prior stroke, baseline NIHSS, ASPECTS, onset to imaging time, and TOAST classification. Furthermore, to examine the associations between CTA imaging characteristics and clinical outcomes in large ischemic stroke, logistic regression was applied for binary outcomes and ordinal regression for ordinal outcomes. All analyses were adjusted for age, sex, diabetes, hypertension, prior stroke, baseline NIHSS, ASPECTS, onset to imaging time, TOAST classification, and EVT. Missing data were handled using multiple imputation by chained equations (mice package). Statistical analyses were performed in R version 4.4.2 (R Foundation for Statistical Computing), and a two-tailed p-value < 0.05 was considered statistically significant.

## Results

### Baseline characteristics

**Fig. 1:**
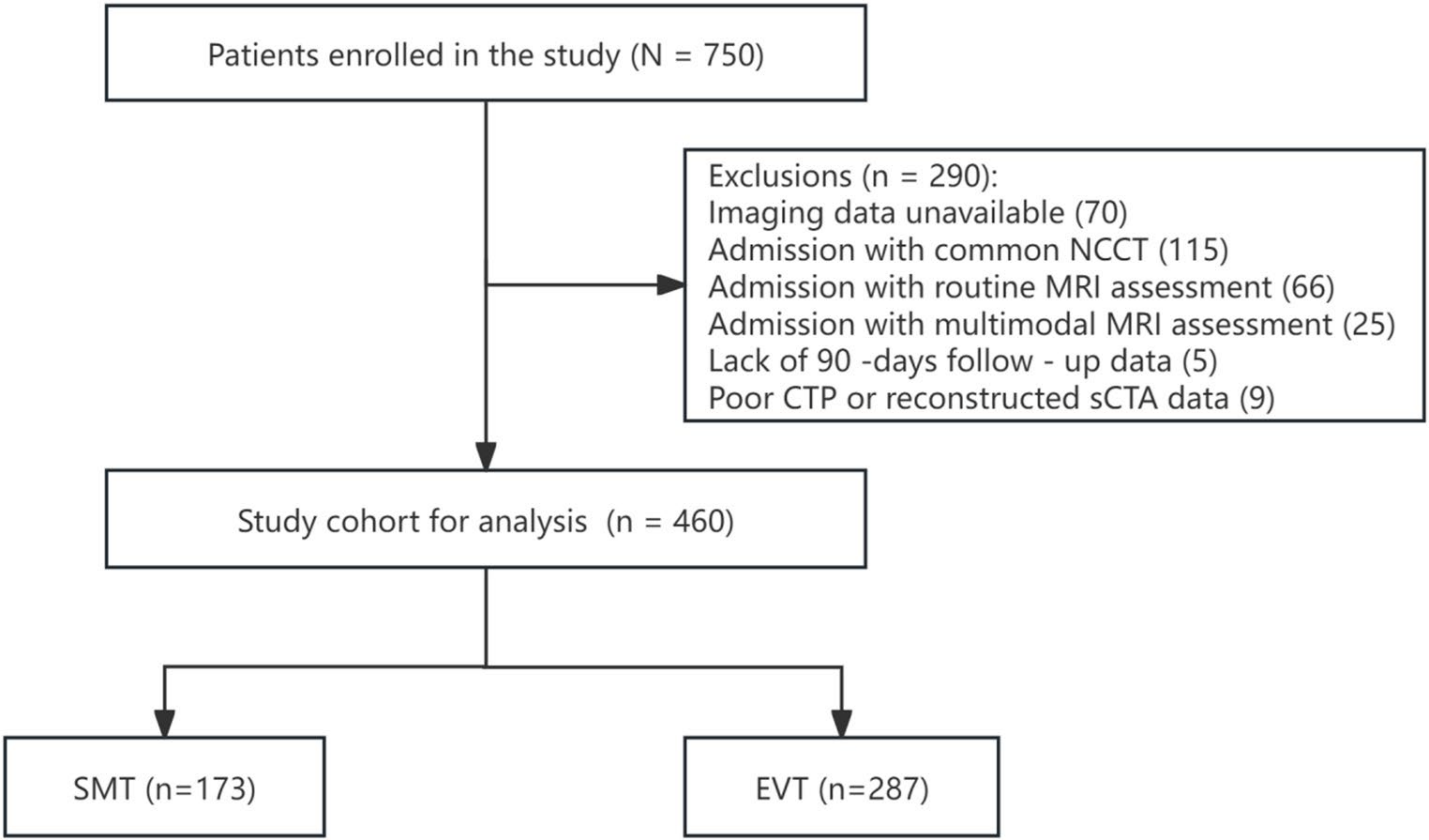
Flowchart of patient selection. SMT, standard medical therapy; EVT, endovascular therapy; NCCT, noncontrast computed tomography; MRI, magnetic resonance imaging; CTA, computed tomography angiography; CTP, computed tomography perfusion

A total of 460 patients (61.3%) from 38 comprehensive stroke centers in China were eligible for secondary analysis (median age, 69.0 years [IQR, 60.0–78.0]; 255 men, 55.4%). Among them, 287 underwent EVT and 173 underwent SMT (Fig. 1). Compared with patients undergoing SMT, those treated with EVT were younger, had a lower prevalence of hypertension, were more likely to have hyperlipidemia, and presented with higher median ASPECTS (*P* < 0.05). Significant differences were observed between groups in TOAST classification, occlusion site, and clot burden score (all *P* ≤ 0.002). Specifically, cardioembolism (CE), ICA occlusion, and lower clot burden score (0–3) were more frequent in the EVT group, whereas large artery atherosclerosis (LAA), MCA-M1 occlusion, and intermediate clot burden score (4–6) were more common in the SMT group. Other baseline characteristics were well balanced between groups (Table 1). Figures S1 and S2 illustrate the distribution of mean collateral scores (COVES, Tan score, and ICV score) across different occlusion site and clot burden categories, showing lower scores among patients with clot burden score (0–3) and ICA occlusion. Additionally, baseline characteristics stratified by occlusion site, COVES, Tan score, ICV score, and clot burden score are summarized in Tables S1–S5.

**Table 1:** Baseline characteristics of patients undergoing SMT vs. EVT

Variables	Overall (*n* = 460)	SMT (*n* = 173)	EVT (*n* = 287)	*P* value
Patient characteristics				
Age, median (IQR), y	69 (60–78)	72 (65–79)	67 (58–77)	< 0.001
Sex, male, n (%)	255 (55.4)	87 (50.3)	168 (58.5)	0.104
Prior stroke, n (%)	71 (15.4)	32 (18.5)	39 (13.6)	0.201
Smoking, n (%)	144 (31.3)	50 (28.9)	94 (32.8)	0.448
Hypertension, n (%)	295 (64.1)	126 (72.8)	169 (58.9)	0.003
Hyperlipidemia, n (%)	84 (18.3)	23 (13.3)	61 (21.3)	0.044
Diabetes, n (%)	80 (17.4)	34 (19.7)	46 (16.0)	0.386
Atrial fibrillation, n (%)	190 (41.3)	78 (45.1)	112 (39.0)	0.237
Glucose, median (IQR), mmol/l	7.2 (6.1–8.9)	7.1 (6.0–8.5.0.5)	7.3 (6.1–9.1)	0.289
Baseline NIHSS, median (IQR)	17 (14–21)	17 (13–22)	17 (14–21)	0.647
Baseline ASPECTS, median (IQR)	4 (2–5)	3 (1–5)	4 (2–5)	0.003
TOAST classification, n (%)				0.002
LAA	164 (35.7)	79 (45.7)	85 (29.6)	
CE	232 (50.4)	79 (45.7)	153 (53.3)	
Others	64 (13.9)	15 (8.6)	49 (17.1)	
Imaging characteristics				
Occlusion site, n (%)				0.001
ICA	156 (33.9)	40 (23.1)	116 (40.4)	
MCA-M1	246 (53.5)	110 (63.6)	136 (47.4)	
MCA-M2	58 (12.6)	23 (13.3)	35 (12.2)	
Arterial collaterals (Tan 2–3), n (%)	50 (28.9)	70 (24.4)	120 (26.1)	0.338
Superficial venous collaterals (COVES 3–6), n (%)	87 (50.3)	133 (46.3)	220 (47.8)	0.469
Deep venous collaterals (ICV = 1), n (%)	72 (41.6)	117 (40.8)	189 (41.1)	0.935
Clot burden score, n (%)				< 0.001
0–3	132 (28.8)	34 (19.7)	98 (34.3)	
4–6	226 (49.2)	107 (61.8)	119 (41.6)	
7–10	101 (22.0)	32 (18.5)	69 (24.1)	
Treatment characteristics				
Onset to imaging time, median (IQR), min	327 (185–519)	310 (189–513)	342 (183–529)	0.901
IVT, n (%)	129 (28.0)	54 (31.2)	75 (26.1)	0.285

SMT, standard medical therapy; EVT, endovascular therapy; NIHSS, National Institutes of Health Stroke Scale; LAA, large-artery atherosclerosis; CE, cardioembolism; TOAST, Trial of Org10172 in Acute Stroke Treatment; ASPECTS, Acute Stroke Prognosis Early Computed Tomography Score; ICA, internal carotid artery; MCA, middle cerebral artery; COVES, cortical vein opacification score; ICV, internal cerebral veins; IVT, intravenous thrombolysis; mRS, modified Rankin Scale; IQR, interquartile range

### Interaction of CTA Imaging Characteristics With EVT Effect on 90-day mRS

**Fig. 2:**
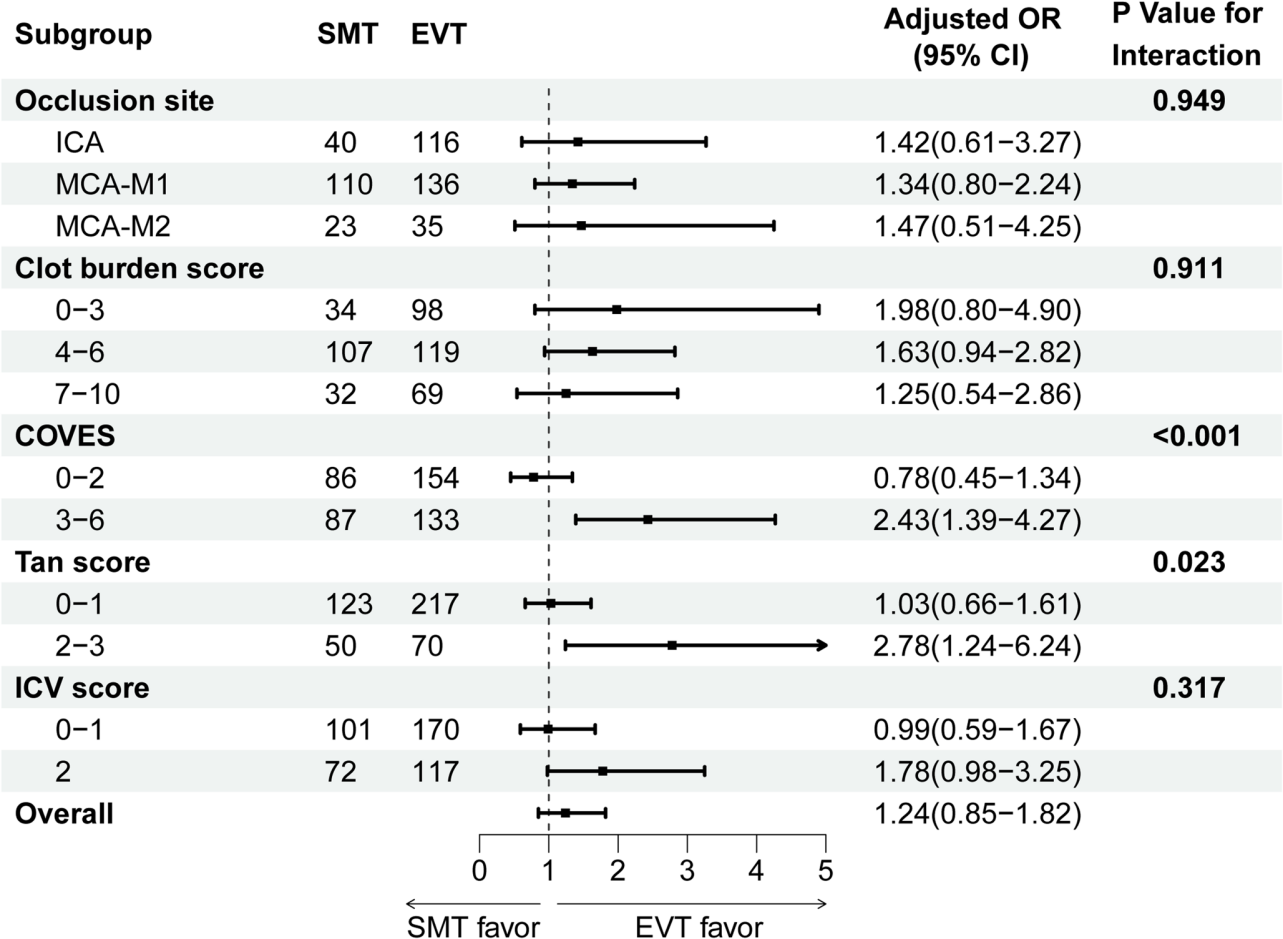
Interaction between CTA imaging characteristics subgroups and treatment modality on the primary outcome. CTA, computed tomography angiography; SMT, standard medical therapy; EVT, endovascular therapy; OR, odds ratio; CI, confidence interval; ICA, internal carotid artery; MCA, middle cerebral artery; COVES, cortical vein opacification score; ICV, internal cerebral veins

The effect of EVT on the 90-day mRS was significantly modified by COVES (*P* < 0.001 for interaction). Among patients with favorable superficial venous collaterals (COVES 3–6), EVT was associated with reduced disability at 90 days (adjusted common odds ratio [acOR], 2.43; 95% CI, 1.39–4.27), whereas no significant association was observed in patients with unfavorable collaterals (COVES 0–2; acOR, 0.78; 95% CI, 0.45–1.34). Similarly, arterial collaterals assessed by Tan score significantly modified the effect of EVT (*P* = 0.023 for interaction). Patients with Tan 2–3 demonstrated a clear treatment benefit (acOR, 2.78; 95% CI, 1.24–6.24), whereas no significant effect was observed among those with Tan 0–1 (acOR, 1.03; 95% CI, 0.66–1.61). The distribution of 90-day mRS, stratified by treatment group and collateral status according to dichotomized COVES and Tan score, is presented in Figures S3 and S4. In contrast, occlusion site, ICV score, and clot burden score did not significantly modify the association between EVT and 90-day mRS (Fig. 2).

### Association of CTA imaging characteristics with clinical outcomes

We present descriptive statistics for the outcome variables and the results of regression analyses in Table 2 (COVES), Table 3 (Tan score), and Supplementary Tables S6–S8 (occlusion site, ICV score, and clot burden score). The primary outcome differed significantly across subgroups stratified by CTA imaging characteristics (*P* < 0.001). Unadjusted analyses demonstrated that higher-strata CTA subgroups was significantly associated with a shift towards better outcomes on the mRS. Consistently, after multivariable adjustment for age, sex, diabetes, hypertension, prior stroke, baseline NIHSS, ASPECTS, onset to imaging time, TOAST classification, and EVT, higher-strata CTA subgroups was independently associated with a shift towards better outcomes on the mRS, with ordinal odds ratios (aOR) of 3.27 (95% CI, 2.26–4.74) for COVES, 2.24 (95% CI, 1.51–3.31) for Tan score, 2.76 (95% CI, 1.50–5.08) for occlusion site, 2.12 (95% CI, 1.49–3.03) for ICV score, and 4.94 (95% CI, 2.92–8.37) for clot burden score.

**Table 2:** Associations of COVES with clinical outcomes in patients with large ischemic stroke

Outcomes	COVES		*P*-value	Unadjusted OR	*P*-value	Adjusted OR	*P*-value
	0–2	3–6		(95% CI)		(95% CI)	
Primary outcome, median (IQR)							
90-day mRS score	6 (4–6)	4 (2–6)	< 0.001	3.62 (2.56–5.14)	< 0.001	3.27 (2.26–4.74)	< 0.001
Secondary outcomes, n (%)							
mRS 0–2	17 (7.1)	59 (26.8)	< 0.001	4.81 (2.70–8.55)	< 0.001	5.24 (2.73–10.07)	< 0.001
mRS 0–3	40 (16.7)	98 (44.5)	< 0.001	4.02 (2.61–6.18)	< 0.001	4.33 (2.61–7.16)	< 0.001
mRS 0–4	74 (30.8)	135 (61.4)	< 0.001	3.56 (2.42–5.24)	< 0.001	3.62 (2.31–5.68)	< 0.001
Any ICH	83 (34.6)	51 (23.2)	0.010	0.57 (0.38–0.86)	0.007	0.60 (0.37–0.95)	0.03
sICH	52 (24.9)	36 (17.6)	0.089	0.64 (0.40–1.04)	0.07	0.65 (0.38–1.11)	0.116
90-day mortality	138 (57.5)	65 (29.5)	< 0.001	0.31 (0.21–0.46)	< 0.001	0.35 (0.22–0.53)	< 0.001

COVES, cortical vein opacification score; mRS, modified Rankin Scale; sICH, symptomatic intracranial hemorrhage; OR, odds ratio; CI, confidence interval; IQR, interquartile range. The adjusted variables included age, sex, history of diabetes and hypertension, prior stroke, baseline NIHSS, ASPECTS, onset to imaging time, TOAST classification, and endovascular therapy

**Table 3:** Associations of Tan score with clinical outcomes in patients with large ischemic stroke

Outcomes	Tan score		*P*-value	Unadjusted OR	*P*-value	Adjusted OR	*P*-value
	0–1	2–3		(95% CI)		(95% CI)	
Primary outcome, median (IQR)							
90-day mRS score	6 (4–6)	4 (2–6)	< 0.001	2.48 (1.70–3.62)	< 0.001	2.24 (1.51–3.31)	< 0.001
Secondary outcomes, n (%)							
mRS 0–2	44 (12.9)	32 (26.7)	0.001	2.45 (1.46–4.09)	0.001	2.44 (1.36–4.39)	0.003
mRS 0–3	83 (24.4)	55 (45.8)	< 0.001	2.62 (1.69–4.05)	< 0.001	2.63 (1.58–4.39)	< 0.001
mRS 0–4	137 (40.3)	72 (60.0)	< 0.001	2.22 (1.45–3.40)	< 0.001	2.14 (1.31–3.49)	0.002
Any ICH	106 (31.2)	28 (23.3)	0.131	0.67 (0.42–1.09)	0.105	0.84 (0.49–1.42)	0.505
sICH	66 (22.0)	22 (19.3)	0.641	0.85 (0.49–1.45)	0.549	1.02 (0.56–1.83)	0.959
90-day mortality	170 (50.0)	33 (27.5)	< 0.001	0.38 (0.24–0.60)	< 0.001	0.41 (0.25–0.67)	< 0.001

mRS, modified Rankin Scale; sICH, symptomatic intracranial hemorrhage; OR, odds ratio; CI, confidence interval; IQR, interquartile range. The adjusted variables included age, sex, history of diabetes and hypertension, prior stroke, baseline NIHSS, ASPECTS, onset to imaging time, TOAST classification, and endovascular therapy

For secondary outcomes, with the exception of occlusion site, higher-strata CTA subgroups were associated with a significantly increased likelihood of achieving favorable outcomes, including an mRS of 0–2, 0–3, and 0–4 at 90 days, after multivariable adjustment. Furthermore, higher-strata CTA subgroups were inversely associated with 90-day mortality. Additionally, no significant association was found between Tan score, occlusion site, ICV score, or clot burden score and the risk of any ICH or sICH. Although COVES showed no significant association with sICH, it was associated with the risk of any ICH both in univariable (aOR, 0.57; 95% CI, 0.38–0.86) and multivariable (aOR, 0.60; 95% CI, 0.37–0.95) analyses, with the association slightly attenuated but remaining statistically significant after adjustment.

## Discussion

In this subanalysis of a nationwide prospective cohort study, we comprehensively describe CTA imaging characteristics, examined their interaction with EVT effects, and evaluated their prognostic associations with clinical outcomes in patients with large ischemic stroke. A significant interaction was observed between COVES and Tan score in modifying the association between EVT and outcomes, whereas no such effect modification was seen with occlusion site, ICV score, or clot burden score. Importantly, after multivariable adjustment including EVT, higher-strata CTA subgroups were independently associated with favorable outcomes and mortality at 90 days, without a significant association with either any ICH or sICH. These findings suggest that comprehensive assessment of CTA imaging characteristics may enhance the treatment effect and enable more efficient selection of patients for EVT.

Better collateral status are associated with smaller infarct core volumes, as robust collaterals help maintain the mismatch between the infarct core and the penumbra [23]. Accordingly, patient selection based on collateral status has long been a central focus of research in identifying candidates most likely to benefit from EVT. CTA is commonly used to identify patients with poor collaterals, and the Endovascular therapy for ischemic stroke (ESCAPE) trial specifically excluded those with inadequate collaterals from EVT [24]. Nevertheless, the value of CTA-based collateral assessment in guiding EVT selection remains controversial. In a post hoc analysis of MR CLEAN [25], arterial collaterals assessed by baseline CTA (Tan score) was found to modify the treatment effect of EVT. Similarly, the MR CLEAN-LATE trial suggested that Tan score modified the association between EVT and outcomes, with a greater treatment effect observed in patients with poor collaterals (Tan score 1) [9]. In contrast, the Highly Effective Reperfusion Using Multiple Endovascular Devices (HERMES) collaboration, which evaluated baseline imaging in EVT-eligible patients, reported no significant interaction between Tan score and treatment outcomes [12]. In this study, statistically significant interactions were observed between Tan score and the effect of EVT on outcomes, with better arterial collaterals (Tan score 2–3) associated with greater treatment benefit. This finding is inconsistent with the results from MR CLEAN-LATE and HERMES, which may be attributable to the inclusion of patients with small-to-moderate infarcts (ASPECTS 6–10) in the prior studies. Recently, a secondary analysis of the Efficacy and Safety of Thrombectomy in Stroke with Extended Lesion and Extended Time Window (TENSION) trial demonstrated that EVT benefits patients with large ischemic strokes, with no significant interaction between arterial collaterals and treatment effect [26]. This discrepancy with our study may be attributable to the inclusion criteria (ASPECTS 3–5 and onset within 12 h) or to the sample size.

To date, evidence regarding whether venous collaterals modifies the effect of EVT in LVO patients is scarce, particularly among those with large ischemic stroke. In this study, we observed statistically significant interactions between COVES and EVT outcomes, while no such interaction was found for ICV score. However, patients with ICV score of 1 seemed to derive a potential benefit from EVT. Winkelmeier et al. were the first to report that higher COVES were associated with favorable functional outcomes in LVO patients with extensive baseline infarction (ASPECTS ≤ 5) [27]. In the late time window, evidence suggests that favorable venous collaterals (COVES 4–6) are associated with a higher likelihood of achieving functional independence in LVO patients undergoing EVT [17]. These results may be attributed to the interplay between venous drainage, tissue microperfusion, hemorrhagic transformation, infarct growth, and cerebral edema formation. Higher venous opacification suggests better microcirculation in the affected tissue, indicating that more blood is actually reaching the ischemic brain. Furthermore, favorable venous collaterals have been found to correlate strongly with reduced hemorrhagic transformation, infarct growth, and cerebral edema formation after EVT [15–17, 28–30]. Accordingly, it is reasonable to conclude that favorable venous collaterals are closely associated with improved clinical outcomes and may modify the effect of EVT. Notably, the absence of statistically significant effects in the unfavorable collateral subgroup, coupled with the limitations of this subanalysis, prevents drawing robust conclusions. Therefore, we do not recommend excluding any additional patient groups from EVT at this stage, and further research is needed.

We found no significant interactions between occlusion site or clot burden score and EVT outcomes, which is consistent with prior reports [12, 31]. Clots located in the ICA and MCA-M1 segments are generally more accessible to current EVT techniques than those in the MCA-M2 segment. Moreover, ICA and MCA-M1 clots typically present with a greater clot burden than M2 clots. However, these anatomical and volumetric differences do not appear to substantially modify the treatment effect of EVT. In this study, no significant benefit of EVT over SMT was observed in the ICA subgroup, which contrasts with previous reports [12, 31]. This discrepancy may be attributable to the relatively small sample size within this subgroup. Several meta-analyses suggest that EVT for M2 occlusions may confer overall benefit despite higher procedural risks [32, 33]. However, no clear advantage has been demonstrated in patients with mild or more distal occlusions, reflecting ongoing uncertainty in this subgroup, likely due to lower stroke severity and smaller perfusion deficits compared with LVOs [34, 35]. Conversely, intra- and peri-procedural complications may be more frequent owing to the technical challenges of navigating smaller, more delicate vessels [35]. Furthermore, after adjusting for EVT and other factors, we found that more distal arterial segments (MCA-M1 or M2) and lower clot burden (0–3) were associated with better clinical outcomes in patients with large ischemic stroke compared with ICA occlusion and higher clot burden. Therefore, these imaging characteristics should be considered as supplementary factors to ASPECTS-based selection when making treatment decisions for large ischemic stroke in the future.

This study has some limitations. Firstly, as an observational study, some biases were inevitable. However, to minimize bias, we provided training for each center prior to the study and collected prospective data from multiple centers. Second, the imaging data were acquired using different CTA equipment and parameters. However, to ensure consistency, all imaging data were evaluated by an independent core imaging laboratory, where three investigators, blinded to the clinical outcomes, independently assessed the CTA images. Finally, collateral assessment was performed using single-phase CTA, which may overestimate or underestimate collateral status depending on parameters like bolus timing and contrast volume. While multiphase CTA could improve collateral evaluation by providing temporal data, single-phase CTA remains advantageous due to its wide availability and lower cost, both essential for the clinical application of these findings [36].

In conclusion, this subanalysis of a prospective cohort study demonstrates that better arterial and venous collaterals are associated with favorable outcomes and may modify the effect of EVT in patients with large ischemic stroke. Notably, patients with poor collaterals may derive limited benefit from EVT. These findings provide preliminary evidence supporting the utility of baseline CTA imaging in guiding EVT selection for this population.

## Supplementary Information

Supplementary Material 1

## Data Availability

Data generated or analyzed during the study are available from the corresponding author by request.
