# Supplementary material for: CTA imaging characteristics and endovascular therapy effect in patients with large ischemic stroke: a prospective cohort study
Source: Neurol Res Pract. 2026 Apr 17;8(1):25. doi: 10.1186/s42466-026-00492-6 (PMC13091254; doi:10.1186/s42466-026-00492-6)
Supplement: Supplementary file 1 — Supplementary Material 1 [file 42466_2026_492_MOESM1_ESM.docx]

**Supplementary materials**

**Figure S1.** The distribution of mean collateral scores across different occlusion sites


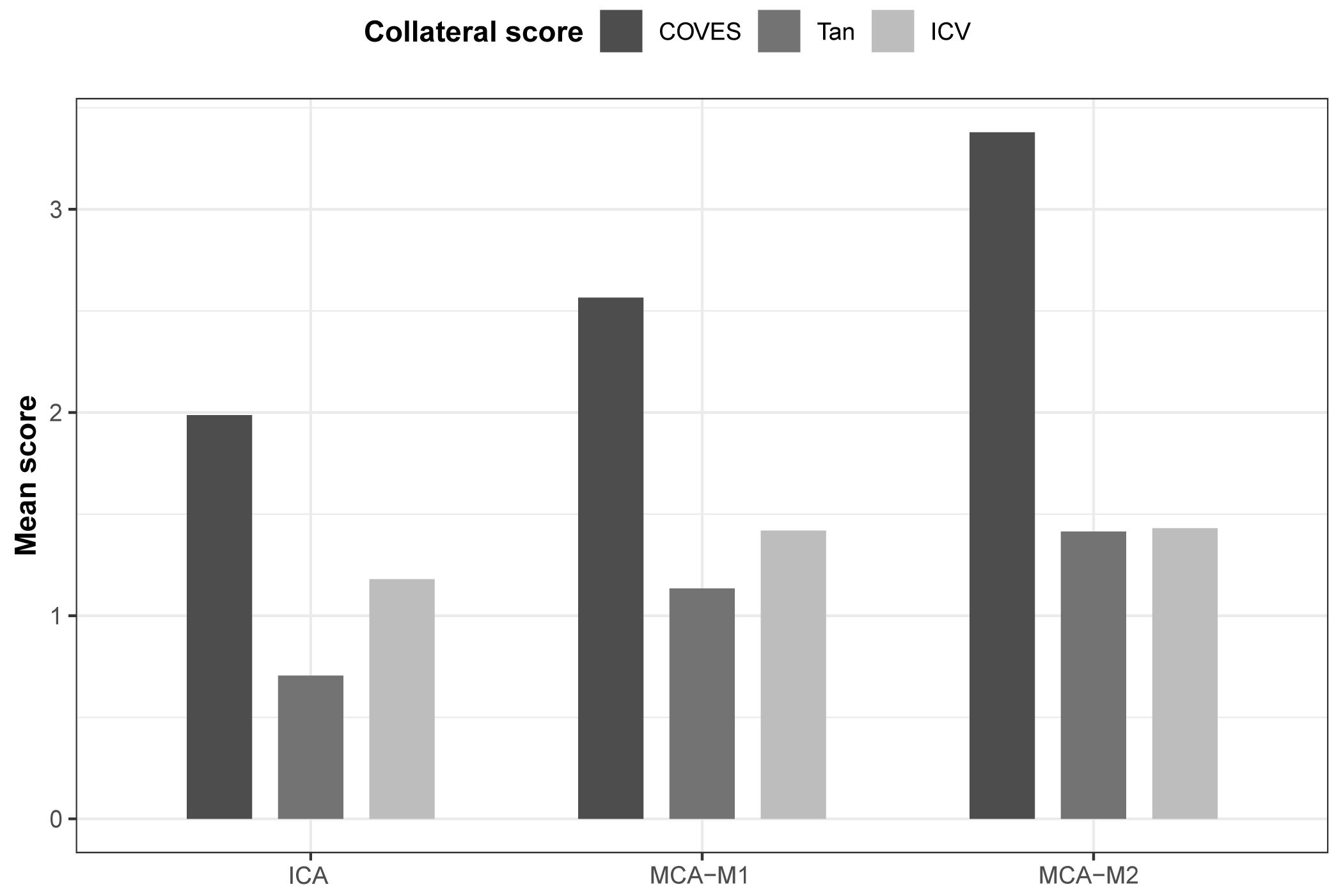


Abbreviations: ICA, internal carotid artery; MCA, middle cerebral artery; COVES, cortical vein opacification score; ICV, internal cerebral veins.

**Figure S2.** The distribution of mean collateral scores across different clot burden score


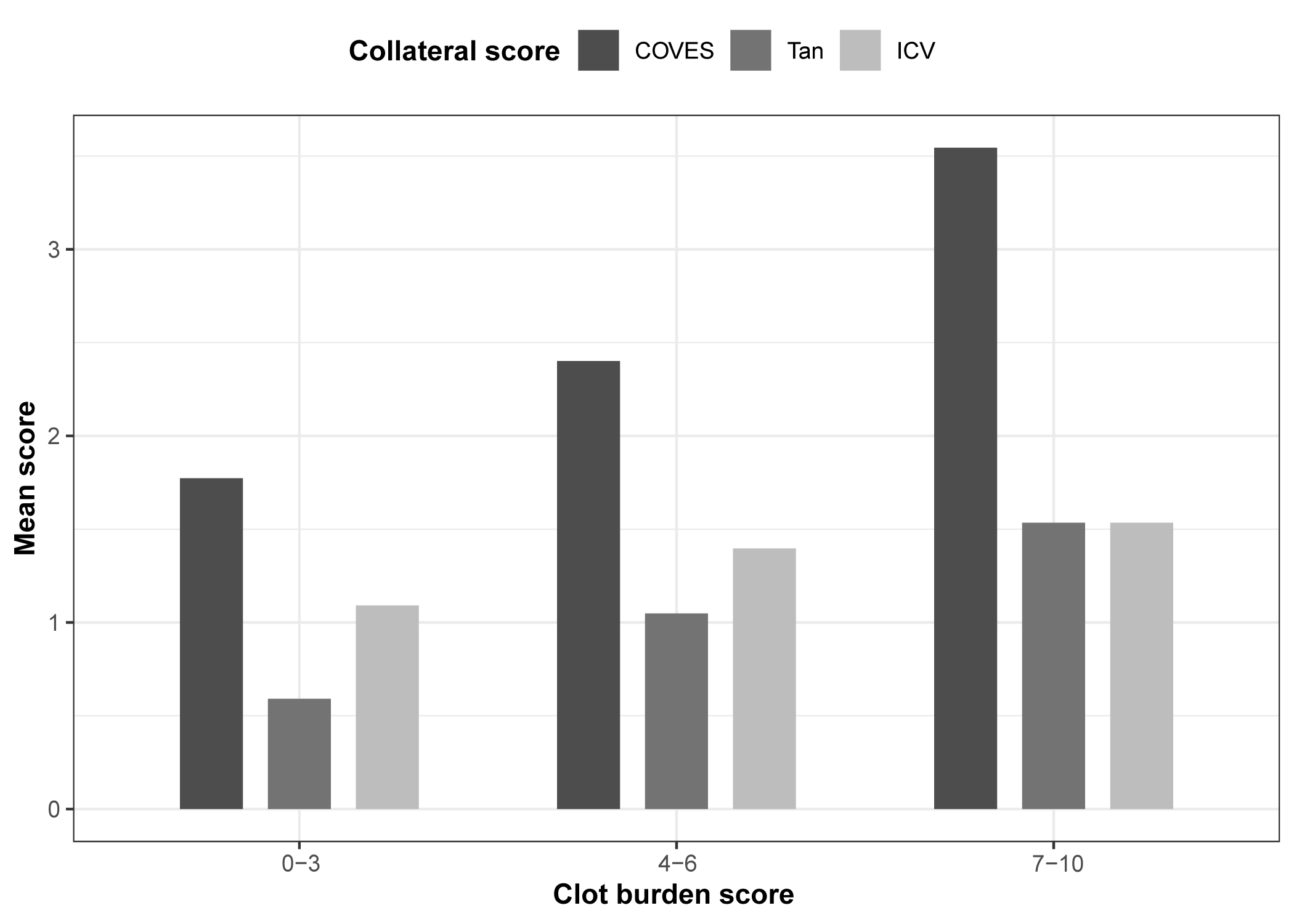


Abbreviations: COVES, cortical vein opacification score; ICV, internal cerebral veins.

**Figure S3.** Distribution of the 90-day mRS according to SMT vs EVT in COVES subgroup


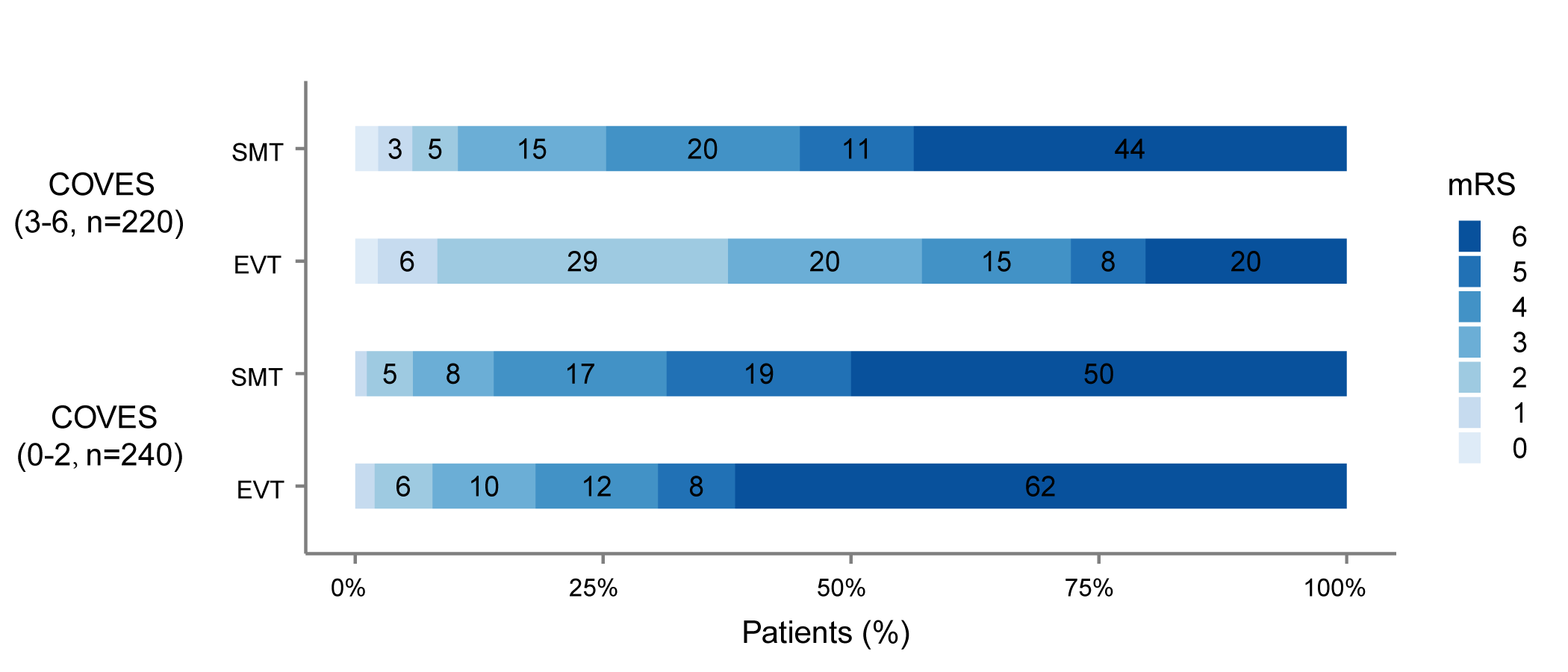


Abbreviations: COVES, cortical vein opacification score; SMT, standard medical therapy; EVT, endovascular therapy; mRS, modified Rankin Scale.

**Figure S4.** Distribution of the 90-day mRS according to SMT vs EVT in Tan score subgroup


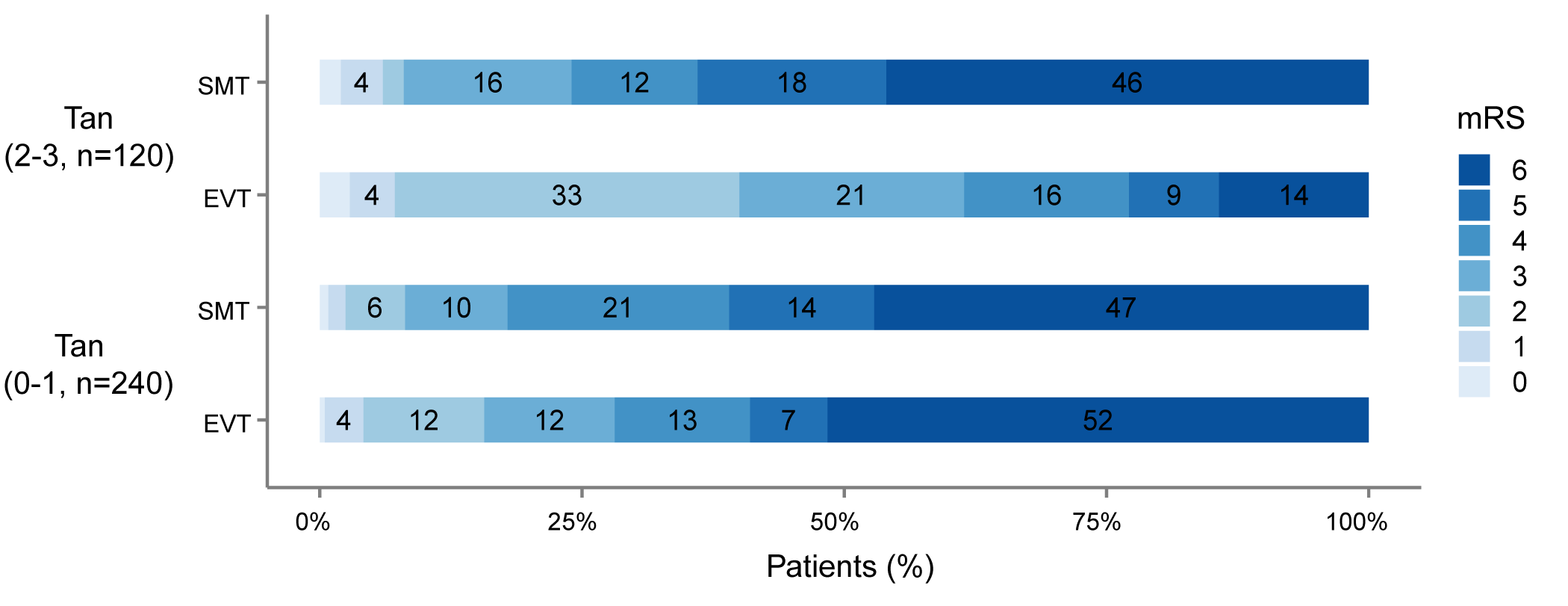


Abbreviations: SMT, standard medical therapy; EVT, endovascular therapy; mRS, modified Rankin Scale.

**Table S1.** Baseline characteristics of the cohort stratified by occlusion site in patients with large ischemic stroke

| **Variables** | **Overall (n=460)** | **ICA (n=156)** | **MCA-M1 (n=246)** | **MCA-M2 (n=58)** | ***P*‐value** |
| --- | --- | --- | --- | --- | --- |
| Patient characteristics |  |  |  |  |  |
| Age, median (IQR), y | 69 (60-78) | 69 (60-77) | 69 (59-77) | 72 (64-81) | 0.123 |
| Sex, male, n (%) | 255 (55.4) | 94 (60.3) | 137 (55.7) | 24 (41.4) | 0.047 |
| Prior stroke, n (%) | 71 (15.4) | 29 (18.6) | 32 (13.0) | 10 (17.2) | 0.295 |
| Smoking, n (%) | 144 (31.3) | 49 (31.4) | 85 (34.6) | 10 (17.2) | 0.038 |
| Hypertension, n (%) | 295 (64.1) | 87 (55.8) | 172 (69.9) | 36 (62.1) | 0.015 |
| Hyperlipidemia, n (%) | 84 (18.3) | 26 (16.7) | 50 (20.3) | 8 (13.8) | 0.418 |
| Diabetes, n (%) | 80 (17.4) | 26 (16.7) | 46 (18.7) | 8 (13.8) | 0.646 |
| Atrial fibrillation, n (%) | 190 (41.3) | 77 (49.4) | 89 (36.2) | 24 (41.4) | 0.033 |
| Glucose, median (IQR), mmol/l | 7.2 (6.1- 8.9) | 7.6 (6.7-9.5) | 7.1 (6.0-8.6) | 6.7 (5.7-8.5) | 0.02 |
| Baseline NIHSS, median (IQR) | 17 (14-21) | 18 (15-23) | 16 (13-20) | 17 (13-20) | 0.011 |
| Baseline ASPECTS, median (IQR) | 4 (2-5) | 3 (1-4) | 4 (2-5) | 5 (4-5) | <0.001 |
| TOAST classification, n (%) |  |  |  |  | 0.015 |
| LAA | 164 (35.7) | 38 (24.4) | 105 (42.7) | 21 (36.2) |  |
| CE | 232 (50.4) | 92 (59.0) | 113 (45.9) | 27 (46.6) |  |
| Others | 64 (13.9) | 26 (16.7) | 28 (11.4) | 10 (17.2) |  |
| Imaging characteristics |  |  |  |  |  |
| Arterial collaterals (Tan 2-3), n (%) | 120 (26.1) | 25 (16.0) | 66 (26.8) | 29 (50.0) | <0.001 |
| Superficial venous collaterals (COVES 3-6), n (%) | 220 (47.8) | 55 (35.3) | 122 (49.6) | 43 (74.1) | <0.001 |
| Deep venous collaterals (ICV=1), n (%) | 189 (41.1) | 42 (26.9) | 118 (48.0) | 29 (50.0) | 0.001 |
| Clot burden score, median (IQR) | 5 (3-6) | 3 (1-4) | 6 (4-6) | 8 (6-9) | <0.001 |
| Treatment characteristics |  |  |  |  |  |
| Onset to imaging time, median (IQR), min | 327 (185-519) | 306 (161-524) | 335 (192-515) | 346 (189-529) | 0.400 |
| IVT, n (%) | 129 (28.0) | 35 (22.4) | 72 (29.3) | 22 (37.9) | 0.066 |

Abbreviations: SMT, standard medical therapy; EVT, endovascular therapy; NIHSS, National Institutes of Health Stroke Scale; LAA, large-artery atherosclerosis; CE, cardioembolism; TOAST, Trial of Org10172 in Acute Stroke Treatment; ASPECTS, Acute Stroke Prognosis Early Computed Tomography Score; COVES, cortical vein opacification score; ICV, internal cerebral veins; IVT, intravenous thrombolysis; mRS, modified Rankin Scale; IQR, interquartile range.

**Table S2.** Baseline characteristics of the cohort stratified by clot burden score in patients with large ischemic stroke

| **Variables** | **Overall (n=460)** | **0-3 (n=132)** | **4-6 (n=226)** | **7-10 (n=101)** | ***P*‐value** |
| --- | --- | --- | --- | --- | --- |
| Patient characteristics |  |  |  |  |  |
| Age, median (IQR), y | 69 (60-78) | 69 (63-77) | 69 (60-78) | 69 (59-79) | 0.987 |
| Sex, male, n (%) | 255 (55.4) | 79 (59.8) | 123 (54.4) | 53 (52.5) | 0.475 |
| Prior stroke, n (%) | 71 (15.4) | 23 (17.4) | 37 (16.4) | 11 (10.9) | 0.342 |
| Smoking, n (%) | 144 (31.3) | 43 (32.6) | 72 (31.9) | 29 (28.7) | 0.800 |
| Hypertension, n (%) | 295 (64.1) | 78 (59.1) | 163 (72.1) | 53 (52.5) | 0.001 |
| Hyperlipidemia, n (%) | 84 (18.3) | 27 (20.5) | 42 (18.6) | 15 (14.9) | 0.542 |
| Diabetes, n (%) | 80 (17.4) | 21 (15.9) | 41 (18.1) | 18 (17.8) | 0.860 |
| Atrial fibrillation, n (%) | 190 (41.3) | 61 (46.2) | 89 (39.4) | 40 (39.6) | 0.412 |
| Glucose, median (IQR), mmol/l | 7.2 (6.1- 8.9) | 7.6 (6.5-9.1) | 7.2 (6.1-8.9) | 6.7 (5.8-8.5) | 0.064 |
| Baseline NIHSS, median (IQR) | 17 (14-21) | 18 (15-22) | 17 (14-21) | 15 (13-19) | 0.002 |
| Baseline ASPECTS, median (IQR) | 4 (2-5) | 3 (1-4) | 4 (2-5) | 5 (3-5) | <0.001 |
| TOAST classification, n (%) |  |  |  |  | 0.179 |
| LAA | 164 (35.7) | 36 (27.3) | 93 (41.2) | 35 (34.7) |  |
| CE | 232 (50.4) | 75 (56.8) | 105 (46.5) | 51 (50.5) |  |
| Others | 64 (13.9) | 21 (16.9) | 28 (12.4) | 15 (14.8) |  |
| Imaging characteristics |  |  |  |  |  |
| Arterial collaterals (Tan 2-3), n (%) | 120 (26.1) | 9 (6.8) | 55 (24.3) | 56 (55.5) | <0.001 |
| Superficial venous collaterals (COVES 3-6), n (%) | 220 (47.8) | 37 (28.0) | 100 (44.2) | 83 (82.2) | <0.001 |
| Deep venous collaterals (ICV=1), n (%) | 189 (41.1) | 30 (22.7) | 102 (45.1) | 57 (56.4) | <0.001 |
| Treatment characteristics |  |  |  |  |  |
| Onset to imaging time, median (IQR), min | 327 (185-519) | 349 (176-512) | 316 (186-520) | 341 (190-518) | 0.940 |
| IVT, n (%) | 129 (28.0) | 25 (18.9) | 69 (30.5) | 35 (34.7) | 0.016 |

Abbreviations: SMT, standard medical therapy; EVT, endovascular therapy; NIHSS, National Institutes of Health Stroke Scale; LAA, large-artery atherosclerosis; CE, cardioembolism; TOAST, Trial of Org10172 in Acute Stroke Treatment; ASPECTS, Acute Stroke Prognosis Early Computed Tomography Score; ICA, internal carotid artery; MCA, middle cerebral artery; COVES, cortical vein opacification score; ICV, internal cerebral veins; IVT, intravenous thrombolysis; mRS, modified Rankin Scale; IQR, interquartile range.

**Table S3.** Baseline characteristics of the cohort stratified by COVES in patients with large ischemic stroke

| **Variables** | **Overall (n=460)** | **0-2 (n=240)** | **3-6 (n=220)** | ***P*‐value** |  |
| --- | --- | --- | --- | --- | --- |
| Patient characteristics | |  |  |  |  |
| Age, median (IQR), y | | 69 (60-78) | 69 (61-78) | 69 (59-77) | 0.318 |
| Sex, male, n (%) | | 255 (55.4) | 124 (51.7) | 131 (59.5) | 0.109 |
| Prior stroke, n (%) | | 71 (15.4) | 39 (16.2) | 32 (14.5) | 0.707 |
| Smoking, n (%) | | 144 (31.3) | 72 (30.0) | 72 (32.7) | 0.596 |
| Hypertension, n (%) | | 295 (64.1) | 166 (69.2) | 129 (58.6) | 0.024 |
| Hyperlipidemia, n (%) | | 84 (18.3) | 42 (17.5) | 42 (19.1) | 0.749 |
| Diabetes, n (%) | | 80 (17.4) | 44 (18.3) | 36 (16.4) | 0.665 |
| Atrial fibrillation, n (%) | | 190 (41.3) | 102 (42.5) | 88 (40.0) | 0.653 |
| Glucose, median (IQR), mmol/l | | 7.2 (6.1- 8.9) | 7.6 (6.2-9.5) | 7.0 (5.8-8.2) | 0.001 |
| Baseline NIHSS, median (IQR) | | 17 (14-21) | 18 (14-22) | 16 (13-20) | 0.001 |
| Baseline ASPECTS, median (IQR) | | 4 (2-5) | 3 (1-5) | 4 (2-5) | 0.002 |
| TOAST classification, n (%) | |  |  |  | 0.760 |
| LAA | | 164 (35.7) | 88 (36.7) | 76 (34.5) |  |
| CE | | 232 (50.4) | 117 (48.8) | 115 (52.3) |  |
| Others | | 64 (13.9) | 35 (14.6) | 29 (13.1) |  |
| Imaging characteristics | |  |  |  |  |
| Occlusion site, n (%) | |  |  |  | <0.001 |
| ICA | | 156 (33.9) | 101 (42.1) | 55 (25.0) |  |
| MCA-M1 | | 246 (53.5) | 124 (51.7) | 122 (55.5) |  |
| MCA-M2 | | 58 (12.6) | 15 (6.2) | 43 (19.5) |  |
| Arterial collaterals (Tan 2-3), n (%) | | 120 (26.1) | 11 (4.6) | 109 (49.6) | <0.001 |
| Deep venous collaterals (ICV=1), n (%) | | 189 (41.1) | 68 (28.3) | 121 (55.0) | <0.001 |
| Clot burden score, median (IQR) | | 5 (3-6) | 4 (2-6) | 6 (4-8) | <0.001 |
| Treatment characteristics | |  |  |  |  |
| Onset to imaging time, median (IQR), min | | 327 (185-519) | 305 (163-455) | 369 (202-587) | 0.001 |
| IVT, n (%) | | 129 (28.0) | 70 (29.2) | 59 (26.8) | 0.648 |

Abbreviations: SMT, standard medical therapy; EVT, endovascular therapy; NIHSS, National Institutes of Health Stroke Scale; LAA, large-artery atherosclerosis; CE, cardioembolism; TOAST, Trial of Org10172 in Acute Stroke Treatment; ASPECTS, Acute Stroke Prognosis Early Computed Tomography Score; ICA, internal carotid artery; MCA, middle cerebral artery; COVES, cortical vein opacification score; ICV, internal cerebral veins; IVT, intravenous thrombolysis; mRS, modified Rankin Scale; IQR, interquartile range.

**Table S4.** Baseline characteristics of the cohort stratified by Tan score in patients with large ischemic stroke

|  | **Variables** | **Overall (n=460)** | **0-1 (n=340)** | **2-3 (n=120)** | ***P*‐value** |
| --- | --- | --- | --- | --- | --- |
| Patient characteristics | |  |  |  |  |
| Age, median (IQR), y | | 69 (60-78) | 70 (61-77) | 68 (58-80) | 0.629 |
| Sex, male, n (%) | | 255 (55.4) | 186 (54.7) | 69 (57.5) | 0.673 |
| Prior stroke, n (%) | | 71 (15.4) | 53 (15.6) | 18 (15.0) | 0.995 |
| Smoking, n (%) | | 144 (31.3) | 111 (32.6) | 33 (27.5) | 0.352 |
| Hypertension, n (%) | | 295 (64.1) | 219 (64.4) | 76 (63.3) | 0.919 |
| Hyperlipidemia, n (%) | | 84 (18.3) | 58 (17.1) | 26 (21.7) | 0.324 |
| Diabetes, n (%) | | 80 (17.4) | 55 (16.2) | 25 (20.8) | 0.309 |
| Atrial fibrillation, n (%) | | 190 (41.3) | 148 (43.5) | 42 (35.0) | 0.128 |
| Glucose, median (IQR), mmol/l | | 7.2 (6.1- 8.9) | 7.4 (6.2-9.1) | 6.8 (5.8-8.3) | 0.009 |
| Baseline NIHSS, median (IQR) | | 17 (14-21) | 18 (14-22) | 16 (13-20) | 0.004 |
| Baseline ASPECTS, median (IQR) | | 4 (2-5) | 3 (2-5) | 4 (3-5) | 0.010 |
| TOAST classification, n (%) | |  |  |  | 0.249 |
| LAA | | 164 (35.7) | 117 (34.4) | 47 (39.2) |  |
| CE | | 232 (50.4) | 177 (52.1) | 55 (45.8) |  |
| Others | | 64 (13.9) | 46 (13.5) | 18 (15.0) |  |
| Imaging characteristics | |  |  |  |  |
| Occlusion site, n (%) | |  |  |  | <0.001 |
| ICA | | 156 (33.9) | 131 (38.5) | 25 (20.8) |  |
| MCA-M1 | | 246 (53.5) | 180 (52.9) | 66 (55.0) |  |
| MCA-M2 | | 58 (12.6) | 29 (8.5) | 29 (24.2) |  |
| Superficial venous collaterals (COVES 3-6), n (%) | | 220 (47.8) | 111 (32.6) | 109 (90.8) | <0.001 |
| Deep venous collaterals (ICV=1), n (%) | | 189 (41.1) | 112 (32.9) | 77 (64.2) | <0.001 |
| Clot burden score, median (IQR) | | 5 (3-6) | 4 (2-6) | 6 (5-8) | <0.001 |
| Treatment characteristics | |  |  |  |  |
| Onset to imaging time, median (IQR), min | | 327 (185-519) | 316 (173-503) | 345 (205-571) | 0.124 |
| IVT, n (%) | | 129 (28.0) | 89 (26.2) | 40 (33.3) | 0.167 |

Abbreviations: SMT, standard medical therapy; EVT, endovascular therapy; NIHSS, National Institutes of Health Stroke Scale; LAA, large-artery atherosclerosis; CE, cardioembolism; TOAST, Trial of Org10172 in Acute Stroke Treatment; ASPECTS, Acute Stroke Prognosis Early Computed Tomography Score; ICA, internal carotid artery; MCA, middle cerebral artery; COVES, cortical vein opacification score; ICV, internal cerebral veins; IVT, intravenous thrombolysis; mRS, modified Rankin Scale; IQR, interquartile range.

**Table S5.** Baseline characteristics of the cohort stratified by ICV score in patients with large ischemic stroke

|  | **Variables** | **Overall (n=460)** | **0-1 (n=271)** | **3 (n=189)** | ***P*‐value** |
| --- | --- | --- | --- | --- | --- |
| Patient characteristics | |  |  |  |  |
| Age, median (IQR), y | | 69 (60-78) | 69 (63-79) | 68 (58-77) | 0.137 |
| Sex, male, n (%) | | 255 (55.4) | 150 (55.4) | 105 (55.6) | 1.000 |
| Prior stroke, n (%) | | 71 (15.4) | 47 (17.3) | 24 (12.7) | 0.220 |
| Smoking, n (%) | | 144 (31.3) | 85 (31.4) | 59 (31.2) | 1.000 |
| Hypertension, n (%) | | 295 (64.1) | 183 (67.5) | 112 (59.3) | 0.085 |
| Hyperlipidemia, n (%) | | 84 (18.3) | 43 (15.9) | 41 (21.7) | 0.142 |
| Diabetes, n (%) | | 80 (17.4) | 47 (17.3) | 33 (17.5) | 1.000 |
| Atrial fibrillation, n (%) | | 190 (41.3) | 115 (42.4) | 75 (39.7) | 0.621 |
| Glucose, median (IQR), mmol/l | | 7.2 (6.1- 8.9) | 7.3 (6.1-9.1) | 7.0 (5.9-8.6) | 0.071 |
| Baseline NIHSS, median (IQR) | | 17 (14-21) | 18 (14-22) | 16 (13-20) | 0.009 |
| Baseline ASPECTS, median (IQR) | | 4 (2-5) | 3 (2-5) | 4 (2-5) | 0.026 |
| TOAST classification, n (%) | |  |  |  | 0.424 |
| LAA | | 164 (35.7) | 99 (36.5) | 65 (34.4) |  |
| CE | | 232 (50.4) | 130 (48.0) | 102 (54.0) |  |
| Others | | 64 (13.9) | 42 (15.5) | 22 (11.6) |  |
| Imaging characteristics | |  |  |  |  |
| Occlusion site, n (%) | |  |  |  | <0.001 |
| ICA | | 156 (33.9) | 114 (42.1) | 42 (22.2) |  |
| MCA-M1 | | 246 (53.5) | 128 (47.2) | 118 (62.4) |  |
| MCA-M2 | | 58 (12.6) | 29 (10.7) | 29 (15.3) |  |
| Superficial venous collaterals (COVES 3-6), n (%) | | 220 (47.8) | 99 (36.5) | 121 (64.0) | <0.001 |
| Arterial collaterals (Tan 2-3), n (%) | | 120 (26.1) | 43 (15.9) | 77 (40.8) | <0.001 |
| Clot burden score, median (IQR) | | 5 (3-6) | 4 (2-6) | 6 (4-7) | <0.001 |
| Treatment characteristics | |  |  |  |  |
| Onset to imaging time, median (IQR), min | | 327 (185-519) | 316 (190-484) | 357 (175-568) | 0.232 |
| IVT, n (%) | | 129 (28.0) | 78 (28.8) | 51 (27.0) | 0.751 |

Abbreviations: SMT, standard medical therapy; EVT, endovascular therapy; NIHSS, National Institutes of Health Stroke Scale; LAA, large-artery atherosclerosis; CE, cardioembolism; TOAST, Trial of Org10172 in Acute Stroke Treatment; ASPECTS, Acute Stroke Prognosis Early Computed Tomography Score; ICA, internal carotid artery; MCA, middle cerebral artery; COVES, cortical vein opacification score; ICV, internal cerebral veins; IVT, intravenous thrombolysis; mRS, modified Rankin Scale; IQR, interquartile range.

**Table S6.** Associations of occlusion site with clinical outcomes in patients with large ischemic stroke

| **Outcomes** | **Occlusion site** | | |  | **ICA vs. MCA-M1** | | | |  | | | **ICA vs. MCA-M2** | | |
| --- | --- | --- | --- | --- | --- | --- | --- | --- | --- | --- | --- | --- | --- | --- |
|  | **ICA** | **MCA-M1** | **MCA-M2** | ***P*‐value** | |  | **Adjusted OR (95% CI)** | ***P*‐value** | |  | **Adjusted OR (95% CI)** | | ***P*‐value** |  |
| Primary outcome, median (IQR) |  |  |  |  | |  |  |  | |  |  | |  |  |
| 90-day mRS score | 6 (4-6) | 4 (3-6) | 4 (3-6) | <0.001 | |  | 2.04 (1.34-3.1) | 0.001 | |  | 2.76 (1.5-5.08) | | 0.001 |  |
| Secondary outcomes , n (%) |  |  |  |  | |  |  |  | |  |  | |  |  |
| mRS 0–2 | 19 (12.2) | 46 (18.7) | 11 (19.0) | 0.199 | |  | 1.83 (0.94-3.56) | 0.075 | |  | 2.07 (0.8-5.36) | | 0.133 |  |
| mRS 0–3 | 36 (23.1) | 81 (32.9) | 21 (36.2) | 0.060 | |  | 1.55 (0.9-2.67) | 0.116 | |  | 2.04 (0.94-4.47) | | 0.073 |  |
| mRS 0–4 | 51 (32.7) | 127 (51.6) | 31 (53.4) | <0.001 | |  | 2.25 (1.36-3.72) | 0.002 | |  | 2.98 (1.42-6.25) | | 0.004 |  |
| Any ICH | 57 (36.5) | 63 (25.6) | 14 (24.1) | 0.042 | |  | 0.83 (0.51-1.36) | 0.469 | |  | 0.70 (0.32-1.50) | | 0.358 |  |
| sICH | 35 (26.1) | 44 (19.4) | 9 (17.0) | 0.229 | |  | 0.92 (0.52-1.64) | 0.785 | |  | 0.81 (0.33-2.00) | | 0.65 |  |
| 90-day mortality | 92 (59.0) | 92 (37.4) | 19 (32.8) | <0.001 | |  | 0.46 (0.28-0.74) | 0.001 | |  | 0.32 (0.15-0.66) | | 0.002 |  |

Abbreviations: ICA, internal carotid artery; MCA, middle cerebral artery; mRS, modified Rankin Scale; sICH, symptomatic intracranial hemorrhage; OR, odds ratio; CI, confidence interval; IQR, interquartile range. The adjusted variables included age, sex, history of diabetes and hypertension, prior stroke, baseline NIHSS, ASPECTS, onset to imaging time, TOAST classification, and endovascular therapy.

**Table S7.** Associations of ICV score with clinical outcomes in patients with large ischemic stroke

| **Outcomes** | **ICV score** | | ***P*‐value** |  | **Unadjusted OR**  **(95% CI)** | ***P*‐value** |  | **Adjusted OR**  **(95% CI)** | ***P*‐value** |
| --- | --- | --- | --- | --- | --- | --- | --- | --- | --- |
|  | **0-1** | **2** |  |  |  |  |  |  |  |
| Primary outcome, median (IQR) |  |  |  |  |  |  |  |  |  |
| 90-day mRS score | 6 (4-6) | 4 (3-6) | <0.001 |  | 2.54 (1.81-3.59) | <0.001 |  | 2.12 (1.49-3.03) | <0.001 |
| Secondary outcomes , n (%) |  |  |  |  |  |  |  |  |  |
| mRS 0–2 | 31 (11.4) | 45 (23.8) | 0.001 |  | 2.42 (1.46-4.00) | 0.001 |  | 2.00 (1.15-3.48) | 0.015 |
| mRS 0–3 | 60 (22.1) | 78 (41.3) | <0.001 |  | 2.47 (1.64-3.71) | <0.001 |  | 2.17 (1.37-3.45) | 0.001 |
| mRS 0–4 | 100 (36.9) | 109 (57.7) | <0.001 |  | 2.33 (1.59-3.41) | <0.001 |  | 2.04 (1.33-3.15) | 0.001 |
| Any ICH | 82 (30.3) | 52 (27.5) | 0.594 |  | 0.87 (0.58-1.32) | 0.524 |  | 0.81 (0.58-1.44) | 0.69 |
| sICH | 47 (19.9) | 41 (23.0) | 0.518 |  | 1.20 (0.75-1.93) | 0.443 |  | 1.26 (0.75-2.13) | 0.385 |
| 90-day mortality | 147 (54.2) | 56 (29.6) | <0.001 |  | 0.36 (0.24-0.53) | <0.001 |  | 0.41 (0.27-0.64) | <0.001 |

Abbreviations: ICV, internal cerebral veins; mRS, modified Rankin Scale; sICH, symptomatic intracranial hemorrhage; OR, odds ratio; CI, confidence interval; IQR, interquartile range. The adjusted variables included age, sex, history of diabetes and hypertension, prior stroke, baseline NIHSS, ASPECTS, onset to imaging time, TOAST classification, and endovascular therapy.

**Table S8.** Associations of clot burden score with clinical outcomes in patients with large ischemic stroke

| **Outcomes** | **Clot burden score** | | | |  | **0-3 vs. 4-6** | |  | **0-3 vs. 7-10** | |
| --- | --- | --- | --- | --- | --- | --- | --- | --- | --- | --- |
|  | **0-3** | **4-6** | **7-10** | ***P*‐value** |  | **Adjusted OR (95% CI)** | ***P*‐value** |  | **Adjusted OR (95% CI)** | ***P*‐value** |
| Primary outcome, median (IQR) |  |  |  |  |  |  |  |  |  |  |
| 90-day mRS score | 6 (4-6) | 5 (3-6) | 3 (2-5) | <0.001 |  | 2.36 (1.5-3.7) | <0.001 |  | 4.94 (2.92-8.37) | <0.001 |
| Secondary outcomes , n (%) |  |  |  |  |  |  |  |  |  |  |
| mRS 0–2 | 12 (9.1) | 35 (15.5) | 29 (28.7) | <0.001 |  | 1.95 (0.91-4.19) | 0.085 |  | 3.6 (1.59-8.16) | 0.002 |
| mRS 0–3 | 20 (15.2) | 67 (29.6) | 51 (50.5) | <0.001 |  | 2.36 (1.35-4.11) | 0.002 |  | 5.71 (3.09-10.57) | <0.001 |
| mRS 0–4 | 37 (28.0) | 105 (46.5) | 67 (66.3) | <0.001 |  | 2.46 (1.44-4.21) | 0.001 |  | 5.23 (2.73-10.03) | <0.001 |
| Any ICH | 49 (37.1) | 55 (24.3) | 29 (28.7) | 0.036 |  | 0.73 (0.43-1.22) | 0.232 |  | 0.84 (0.45-1.57) | 0.59 |
| sICH | 29 (25.9) | 38 (18.2) | 20 (21.7) | 0.267 |  | 0.86 (0.47-1.58) | 0.624 |  | 0.99 (0.48-2.04) | 0.982 |
| 90-day mortality | 83 (62.9) | 96 (42.5) | 23 (22.8) | <0.001 |  | 0.43 (0.26-0.71) | 0.001 |  | 0.18 (0.09-0.35) | <0.001 |

Abbreviations: mRS, modified Rankin Scale; sICH, symptomatic intracranial hemorrhage; OR, odds ratio; CI, confidence interval; IQR, interquartile range. The adjusted variables included age, sex, history of diabetes and hypertension, prior stroke, baseline NIHSS, ASPECTS, onset to imaging time, TOAST classification, and endovascular therapy.
